# RNA interference targeting carbohydrate sulfotransferase 3 diminishes macrophage accumulation, inhibits MMP-9 expression and promotes lung recovery in murine pulmonary emphysema

**DOI:** 10.1186/s12931-015-0310-7

**Published:** 2015-12-09

**Authors:** Yoshiro Kai, Koichi Tomoda, Hiroyuki Yoneyama, Masanori Yoshikawa, Hiroshi Kimura

**Affiliations:** Second Department of Internal Medicine, Nara Medical University, 840 Shijo-cho, Kashihara-city, Nara 634-8521 Japan; Stelic Institute & Co., Inc., 1-9-15 Higashi Azabu, Minato-ku, Tokyo 106-0044 Japan; Department of Internal Medicine, Yoshino-cho National Health Insurance Yoshino Hospital, 130-1 Oaza Tanji, Yoshino-cho, Yoshino-gun, Nara 639-3114 Japan

**Keywords:** Chondroitin sulfate proteoglycans, Chronic obstructive pulmonary disease, Cytokines, Elastase, Macrophages

## Abstract

**Background:**

Chondroitin sulfate proteoglycans are an important mediators in inflammation and leukocyte trafficking. However, their roles in pulmonary emphysema have not been explored.

In a murine model of elastase-induced pulmonary emphysema, we found increased carbohydrate sulfotransferase 3 (CHST3), a specific enzyme that synthesizes chondroitin 6-sulfate proteoglycan (C6SPG). To elucidate the role of C6SPG, we investigated the effect of small interfering RNA (siRNA) targeting CHST3 that inhibits C6SPG-synthesis on the pathogenesis of pulmonary emphysema.

**Methods:**

Mice were intraperitoneally injected with CHST3 siRNA or negative control siRNA on day0 and 7 after intratracheal instillation of elastase. Histology, respiratory function, glycosaminoglycans (GAGs) content, bronchoalveolar lavage (BAL), elastin staining and gene expressions of tumor necrosis factor (TNF)-α and matrix metalloproteinase (MMP)-9 mRNA were evaluated on day7 and/or day21.

**Results:**

CHST3 mRNA increased at day 7 and decreased thereafter in lung. CHST3 siRNA successfully inhibited the expression of CHST3 mRNA throughout the study and this was associated with significant reduction of GAGs and C6SPG. Airway destruction and respiratory function were improved by the treatment with CHST3 siRNA. CHST3 siRNA reduced the number of macrophages both in BAL and lung parenchyma and also suppressed the increased expressions of TNF-α and MMP-9 mRNA. Futhermore, CHST3 siRNA improved the reduction of the elastin in the alveolar walls.

**Conclusions:**

CHST3 siRNA diminishes accumulation of excessive macrophages and the mediators, leading to accelerate the functional recovery from airway damage by repair of the elastin network associated with pulmonary emphysema.

## Background

Cigarette smoke and pollution are primary causes of chronic obstructive pulmonary disease (COPD). COPD is characterized by persistent inflammation in small airways. Emphysema with airspace enlargement due to alveolar destruction is one of the COPD phenotypes [1, 2]. Anti-inflammatory agents which attenuate chronic inflammation and progression in COPD have not been identified yet. Macrophages play a central role in chronic inflammation occurring in COPD by producing various inflammatory mediators, such as tumor necrosis factor (TNF)-α, interleukin (IL)-6, and matrix metalloproteinases (MMPs) [3]. To reduce the chronic inflammation in COPD it is important to attenuate the accumulation of macrophages and the production of these inflammatory cytokines.

Proteoglycans, which are among the major components of the lung cellular matrix, are important mediators in inflammation and leukocyte trafficking. We have reported that deposited chondroitin sulfate proteoglycans (CSPGs) induced recruited macrophages to contribute to chronic inflammation and promote fibrogenesis [4]. On the other hand in patients with mild to moderate COPD, versican, which is known as a large CSPG, was increased [5]. In these contexts, we hypothesize that CSPGs could be crucial in chronic inflammation in COPD as a result of accumulation of macrophages and reduction of deposited CSPGs in the lung may well contribute to attenuate emphysema.

The increased CSPGs in COPD correlate with decreased elastin and forced expiratory volume 1. Elastin is crucial in maintaining elasticity in the lung. In the lung with COPD, elastin fragmentation strongly correlates with decreased airflow obstruction [6]. Elastin-binding protein (EBP) chaperones tropoelastin through the Golgi and endosomal compartments to the cell surface [7]. CSPGs bind to EBP and induce premature shedding of EBP from the cell surface, leading to impaired presentation of tropoelastin, and also thereby inhibit elastin formation [8, 9]. Concerning elastin formation, reduction in deposited CSPGs in the lung also may be useful for attenuating development of COPD.

Carbohydrate sulfotransferase 3 (CHST3) is a specific enzyme that synthesizes chondroitin 6-sulfate, and transfers sulfate to the C-6 position of the N-acetylgalactosamine (GalNAc) residue of chondroitin. CHST3 is the key enzyme that mediates cell migration during axonal growth in both crushed sciatic nerves, brain development and lumbar disc degeneration [10–12]. In addition, mutation in CHST3 results in chondrodysplasia with major involvement of the spine [13]. However, the role of CHST3 in the development of emphysema has not been explored.

RNA interference induced by small interfering RNA (siRNA) has revealed sequence specific gene silencing [14]. Recent reports show atelocollagen-mediated systemic delivery methods for siRNA in various disease models in vivo [15–18]. In the present study to elucidate the role of CHST3 on the development of emphysema, we investigated the effect of siRNA targeting CHST3 on murine emphysema induced by elastase.

## Methods

### Mice

Female C57BL/6 (6-8-week-old) mice were obtained from SLC Japan Inc. (Shizuoka, Japan) and bred in a pathogen-free facility in the laboratory animal research center at Nara Medical University. All procedures performed during these animal experiments were carried out under the control of our committee, in accordance with The Guidelines for Animal Experiments in Nara Medical University, and Guideline Principles for the Care and Use of Laboratory Animals approved by The Japanese Pharmacological Society.

### Animal model

Mice were anesthetized by intraperitoneal injection with sodium pentobarbital (50 mg/kg), and received intratracheally injection of 4 units/50 μL of porcine pancreatic elastase (PPE) (EC134; Elastin Products, Owensville, MO) or saline alone. After PPE injection, mice were intraperitonealy injected with 10 μg CHST3 siRNA (Silencer Pre-designed siRNA: Cat# 16706; Ambion, Austin, TX) or negative control siRNA (Silencer Negative Control siRNA #1: Cat# AM4611; Ambion) with 0.1 % atelocollagen (Koken, Tokyo, Japan) in a 200-μL volume on day 0 and 7. This protocol resulted in the creation of 3 groups: sham mice given negative control siRNA (sham group), PPE mice given negative control siRNA (control group) and PPE mice treated with CHST3 siRNA (CHST3 siRNA group).

### Lung histopathology

The lung was inflated by instilling neutral-buffered 10 % formalin to 25 cm H_2_0. The lung volume was measured as described previously [19, 20]. The tissue was embedded in paraffin, and 4-μm thick sections were stained with hematoxylin and eosin. The mean linear intercept (MLI), a parameter air space enlargement, was calculated by a light microscopy on 20 randomly selected fields as described previously [21].

### Bronchoalveolar lavage

Mice were anesthetized by intraperitoneal injection with sodium pentobarbital (50 mg/kg) and bronchoalveolar lavage fluid (BALF) was collected by flushing the lung via the trachea three times with 0.8 mL of sterile PBS. The cells in the BALF were counted with a hemocytometer and cell differentials were determined in cytospin preparations stained with Diff-Quik products (Baxter, Miami, FL).

### Antibodies and immunohistochemistry

Immunohistochemical staining was performed as previously reported [4, 22]. The following first Abs were used: 3B3 (Seikagaku Corporation, Tokyo, Japan) for paraffin embedded sections, F4/80 (Serotec, Oxford, UK) and CS-56 (Seikagaku Corporation) for frozen sections [23]. 3B3 recognizes an epitope created following chondroitinase ABC (ChABC) degradation of chondroitin-6 sulfate. CS-56 recognizes an epitope on some intact chondroitin sulfate glycosaminoglycan chains. The number of F4/80^+^ cells per mm^2^ of alveolar wall was determined using light microscopy, in at least 30 randomly chosen high-power fields [4]. Immunolabeling for CS-56 and 3B3 were used Histofine kit (Nichirei Bioscience, Tokyo, Japan) according to the manufacturer’s instructions. Tissue sections for 3B3 were pre-incubated in vitro with 0.2 U/mL ChABC (Seikagaku Corporation) in Tris–HCl, pH 8.0, for 1 h at 37 °C.

### Sulfated glycosaminoglycan (GAG) assay

The total GAG content of left lungs, harvested on day 21 after PPE administration, was determined using an Alcian blue-binding assay for the detection of sulphated glycosaminoglycans (WieslabAB, Lund, Sweden) according to the manufacturer’s instructions.

### Quantitative RT-PCR

Total RNA was extracted from the lung sample by homogenization using RNA Iso plus (Takara, Shiga, Japan) and SV total RNA isolation kit (Promega, Madison, WI) and reverse transcribed as previously described [24]. Real-time PCR was performed using real-time PCR DICE and SYBR premix Taq (Takara Bio, Shiga, Japan). To calculate the relative mRNA expression level, the expression of each gene was normalized to that of the reference gene (GAPDH). The primers for real-time RT-PCR were as follows.

Chst3; forward; 5′-TGTTCCTGGCATTTGTGGTCATA-3′,

reverse: ′-CCAACTCGCTCAGGGACAAGA-3′,

TNF-α; forward; 5′- ATGGCCCAGACCCTCACA-3′,

reverse: 5′- GGAGTAGACAAGGTACAACCCATC-3′,

MMP-9; forward; 5′- CCATGCACTGGGCTTAGATCA-3′,

reverse: 5′- GGCCTTGGGTCAGGCTTAGA-3′,

Gapdh; forward; 5′- TGTGTCCGTCGTGGATCTGA-3′,

reverse: 5′- TTGCTGTTGAAGTCGCAGGAG-3′,

### Lung compliance measurements

On day 21 after elastase administration, the mice were anesthetized with ketamine (87 mg/kg) and xylazine (13 mg/kg). A trachea cannula was attached to a FlexiVent (Sireq, Montreal, Quebec, Canada) to analyze lung function. Pressure volume curves were generated, and the static lung compliance was calculated by the FlexiVent software.

### Determination of the elastin content in the alveolar wall

Lung sections were stained with Elastica-van Gieson stain. Stained lung sections were randomly captured three fields (×400) microscopy (DFC280; Leica, Germany). Elastin contents in the sections were quantified using Image J software in blind manner. Images were converted to grey scale. The red channel was thresholded to exclusively reveal the blue-black stained elastic fibers. The total elastin positive pixels were expressed as a percentage of total tissue area as previously described [25].

### Statistical analysis

Differences were evaluated using Student’s *t*-test. *p*-values < 0.05 were considered to indicate statistically significant differences.

## Results

### CHST3 siRNA protects against elastase-induced lung emphysema

**Fig. 1:**
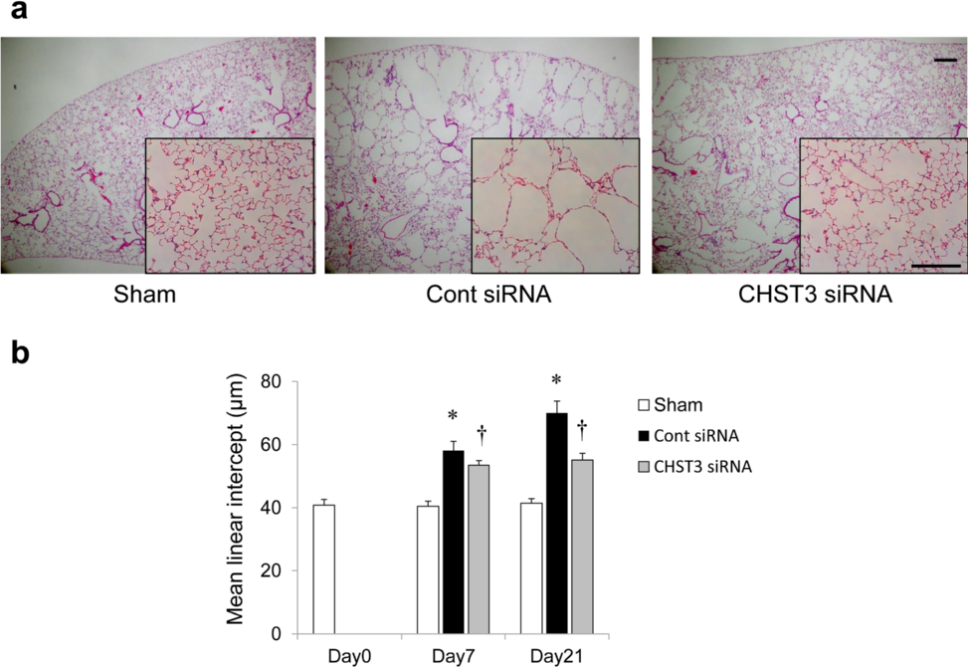
Morphometric assessment after porcine pancreas elastase (PPE) administration. **a** Hematoxylin-eosin (HE) staining of lung tissue 21 days after PPE administration. PPE induced the development of airspace enlargement with destruction of the alveolar walls (Control siRNA). CHST3 siRNA attenuated emphysematous change (CHST3 siRNA). Scale bar 200 μm. **b** Semiquantative analysis of lung tissue using the mean linear intercept (MLI). *n* = 5. *p < 0.01 versus sham group. ^†^ *p* < 0.01 versus control siRNA group

Morphometric assessment was performed on days 7 and 21 after PPE administration. In the control siRNA group, the development of airspace enlargement with progressive destruction of alveolar walls was observed from day 7. However, administration of CHST3 siRNA attenuated the histologic changes in mice given PPE from day 7 (Fig. 1a). We examined the enlargement of the airspaces quantifying the MLI on days 0, 7 and 21. MLI increase was observed in the control siRNA group compared with the sham group from day 7. The increase was significantly attenuated by CHST3 siRNA from day 7 (Fig. 1b).

### Effect of CHST3 siRNA on pulmonary function

**Fig. 2:**
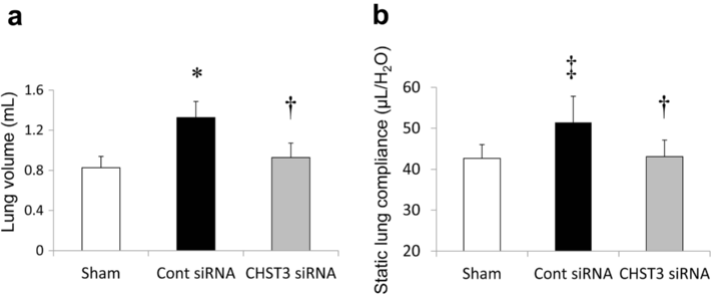
Lung volume and Static lung compliance (Cst) values after PPE administration. **a** Effect of CHST3 siRNA on lung volume 21 days after PPE administration. PPE significantly increased lung volume. CHST3 siRNA suppressed the change. (*n* = 5 per group). *p < 0.01 versus sham group. ^†^ *p* < 0.01 versus control siRNA group. **b** Effect of CHST3 siRNA on lung mechanics day21 after PPE administration. PPE significantly increased Cst. CHST3 siRNA suppressed the change. (*n* = 5 per group). ^‡^ *p* < 0.05 versus sham group. ^†^ *p* < 0.01 versus control siRNA group

The lung volume was significantly increased in the control siRNA on day 21 after PPE administration. CHST3 siRNA significantly attenuated the increase in lung volume (Fig. 2a). To test the effect of CHST3 siRNA on lung dysfunction, static lung compliance (Cst) was assessed. PPE administration significantly enhanced Cst compared with the Sham group. CHST3 siRNA treated mice prevented the increase in Cst (Fig. 2b). These data demonstrate that CHST3 siRNA has a beneficial effect on respiratory function.

### CHST3 siRNA effectively inhibited the expression of CHST3 expression in vivo

**Fig. 3:**
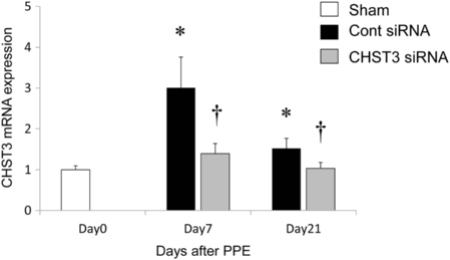
Real time quantitative PCR analysis of CHST3 mRNA in the lung. Total RNA was isolated from lung tissues on days 0, 7 and 21 after PPE administration. (*n* = 5 per group). *p < 0.01 versus the Sham group. ^†^ *p* < 0.01 versus the Control siRNA group

In order to elucidate the role of CHST3 in lung inflammation and emphysema, we examined CHST3 expression on days 0, 7 and 21 after PPE-induced lung injury. After PPE administration, the expression of CHST3 was increased in lung tissue on days 7 and 21. CHST3 siRNA inhibited effectively suppressed the expression of CHST3 on days 7 and 21 (Fig. 3).

### Increased deposition of CSPGs in the elastase-induced emphysema model

**Fig. 4:**
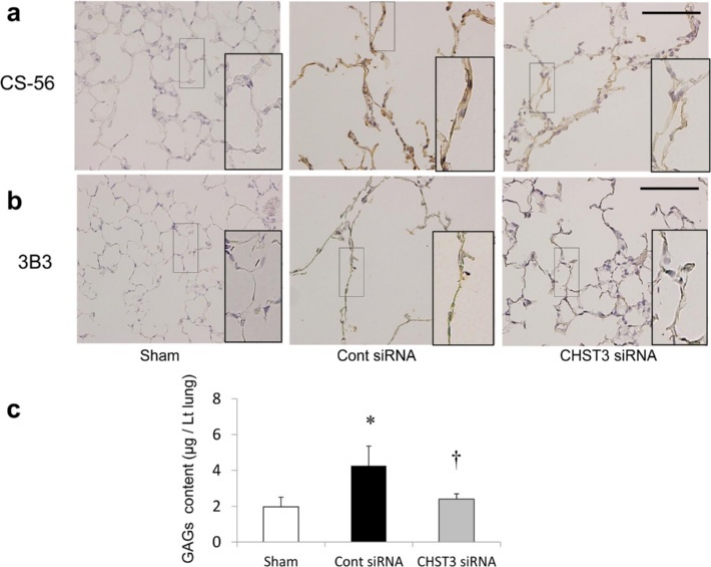
Immunohistochemical detection of CSPGs and Glycosaminoglycans (GAGs) content on days 21 after PPE administration. **a** CS-56 recognizes an epitope on intact chondroitin sulfate glycosaminoglycan chains. CS-56 immunolabeling indicated that CHST3 siRNA treatment suppressed the increased chondroitin sulfate after PPE administration. **b** 3B3 recognizes an epitope created following the degradation of chondroitin-6 sulfate by ChABC. 3B3 immunolabeling indicated that CHST3 siRNA treatment suppressed the increased chondroitin-6 sulfate after PPE administration. Boxes indicate CS-56 or 3B3 staining in alveolar walls. Inserts show high magnification of boxes. Scale bar 100 μm. **c** GAGs content of the left lung on day 21 after PPE administration. (*n* = 5 per group). Mean ± SD, *p < 0.01 versus the Sham group. ^†^ *p* < 0.01 versus the Control siRNA group

We investigated immunohistochemically deposited CSPGs in the injured lung using mAb CS-56 which recognizes an epitope on intact CSPGs chains. CSPGs were barely detectable in normal lungs, but were clearly present on the alveolar wall 21 days after PPE challenge. CHST3 siRNA treatment effectively reduced deposited CSPGs (Fig. 4a). MAb 3B3 recognizes an epitope created following the degradation of chondroitin-6 sulfate by ChABC. Chondroitin 6-sulfate proteoglycan (C6SPG) also increased on alveolar wall after PPE challenge on day 21. However, CHST3 siRNA treatment significantly reduced deposited C6SPG (Fig. 4b).

Deposited GAGs were directly evaluated in the left lungs using a sulphated GAGs assay. In mice treated with PPE, GAGs level had increased 2.2-fold by day 21 compared to the sham group. CHST3 siRNA treatment significantly reduced deposited GAGs, thus confirming its in vivo efficacy (Fig. 4c).

### CHST3 siRNA prevent inflammation after PPE administration

**Fig. 5:**
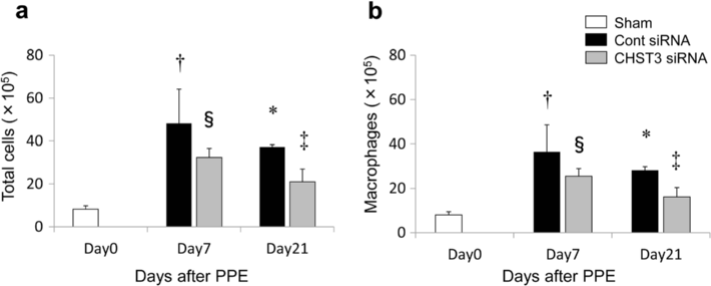
Infiltration of total cells and macrophages in bronchoalveolar lavage fluid (BALF) after PPE administration. CHST3 siRNA treatment significantly reduced the number of (**a**) total cells and (**b**) macrophages in the BALF after PPE administration. (*n* = 5 per group). Mean ± SD, *p < 0.01, ^†^ *p* < 0.05 versus the Sham group. ^‡^ *p* < 0.01 ^§^ *p* < 0.05 versus the Control siRNA group

To investigate the effect of CHST3 siRNA in the inflammatory response to PPE induced injury, we assessed the number of inflammatory cells in the BALF collected from mice after PPE challenge. Intratracheal PPE administration significantly increased the total number of cells and macrophage count in the BALF. CHST3 siRNA treated mice followed a similar pattern, but was significantly reduced in number of the experiment compared with BALF cells from control siRNA treated mice (Fig. 5a, b).

### Inhibited accumulation of F4/80^+^ macrophages by CHST3 siRNA

**Fig. 6:**
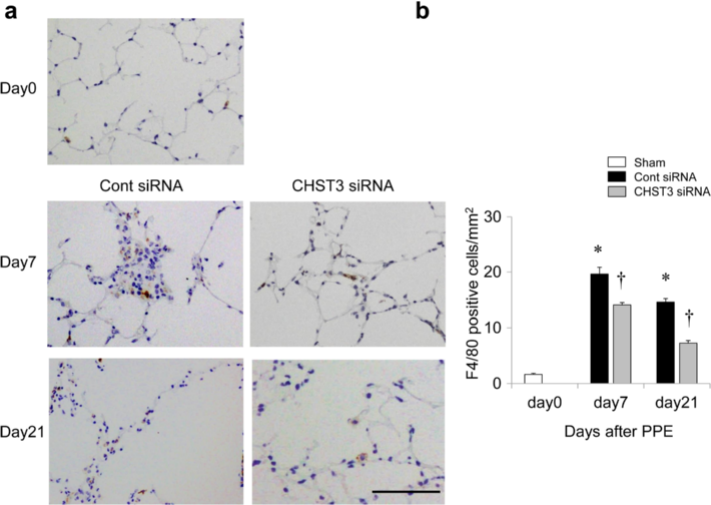
Infiltration of F4/80^+^ macrophages in lung parenchyma after PPE administration. **a** Immunohistochemical detection of F4/80 in untreated lung (d0) or 7 or 21 days after PPE administration. Scale bar 100 μm. **b** Quantitative analysis of F4/80^+^ macrophages in the alveolar area. *p < 0.01 versus the Sham group. ^†^ *p* < 0.01 versus the Control siRNA group

We next investigated the time-course of the appearance of F4/80^+^ macrophages in the lung parenchyma on days 0, 7, and 21. In control siRNA treated mice, a massive accumulation of F4/80^+^ cells was detected on day 7 after PPE challenge and slightly decreased by day 21. However, CHST3 siRNA induced a significantly increased influx of F4/80^+^ cells at all time points (Fig. 6a, b). These data demonstrate that CHST3 siRNA prevents the accumulation of macrophages in the lung parenchyma in response to PPE challenge.

### CHST3 siRNA inhibited TNF-α and MMP-9 expression in vivo after PPE administration

**Fig. 7:**
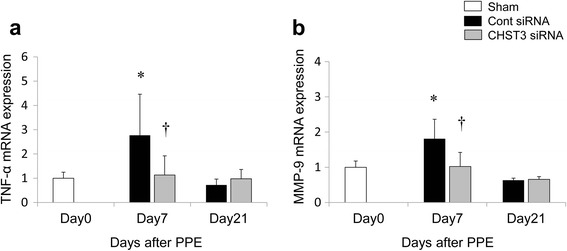
Kinetics of TNF-α and MMP-9 mRNA after PPE administration. Real time quantitative PCR analysis of (**a**) TNF-α, (**b**) MMP-9 mRNA in the lung. Total RNA was isolated from lung tissues on days 0, 7, and 21 after PPE administration. (*n* = 5 per group). *p < 0.05 versus the Sham group. ^†^ *p* < 0.05 versus the Control siRNA group

Since TNF-α and MMP-9 play important roles in the pathogenesis of emphysema, we examined their expressions in lung tissue. The TNF-α and MMP-9 levels were markedly increased in PPE-treated mice on day 7, and treatment with CHST3 siRNA significantly reduced the TNF-α and MMP-9 expression in lung tissues (Fig. 7a, b).

### CHST3 siRNA improved the reduction the elastin of the alveolar walls

**Fig. 8:**
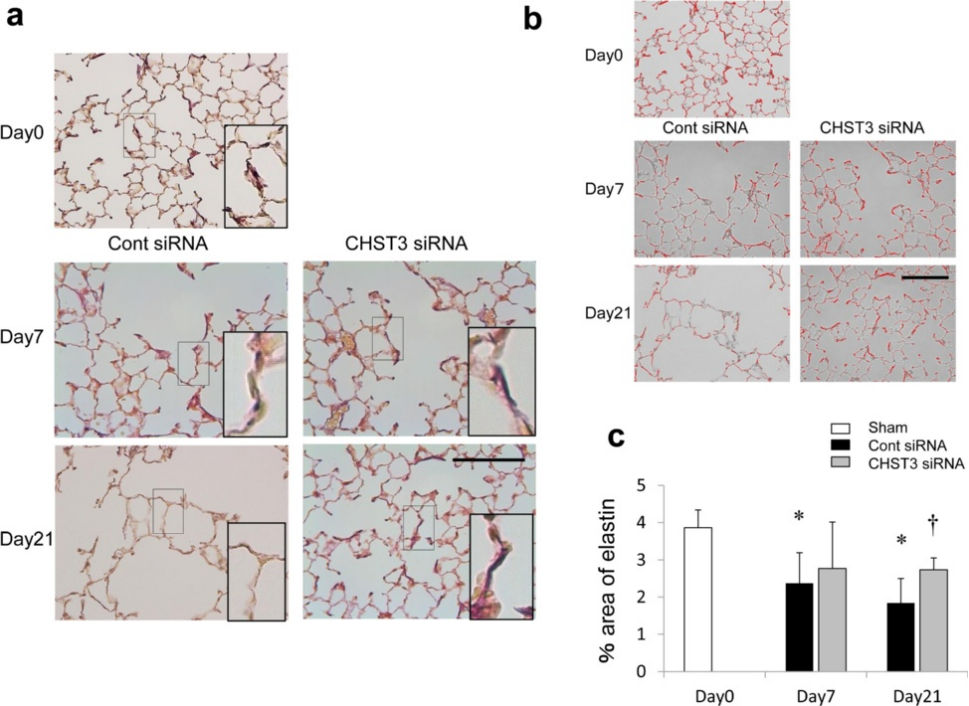
CHST3 siRNA improved the reduction of the elastin in the alveolar walls. **a** Elastica-van Gieson staining of the alveolar wall in untreated lung (d0) or 7 or 21 days after PPE administration. Elastin appeared as distinct blue-black lines through the alveolar walls. Boxes indicate deposition of elastin in alveolar walls. Inserts show high magnification of boxes. Cont siRNA mice on day7 and 21 show discontinuous elastin deposition in walls. CHST3 siRNA mice on day7 and 21 show continuous elastin deposition. Scale bar 100 μm. **b** Images show the area occupied by elastic fibers using imaging software, Image J (red area). **c** Quantification of alveolar wall elastin. Control group mice had significantly lower levels of elastin detected within alveolar walls compared with untreated lung from day 7. CHST3 siRNA treatment suppressed the decreased levels of elastin on day 21. (*n* = 5 per group). *p < 0.01 versus the Sham group. ^†^ *p* < 0.01 versus the Control siRNA group

Elastica-van Gieson staining showed reduced elastin staining in the alveolar wall of control group lung compared with normal lung (Fig. 8a). Quantification was performed using Image J software by measuring % elastin area of alveolar walls. Control group mice had significantly lower levels of elastin detected within alveolar wall compared with normal mice from day 7. CHST3 siRNA treatment suppressed the decreased of % area of elastin on day21 (Fig. 8b, c).

## Discussion

Alveolar macrophages play a pivotal role in the pathophysiology of COPD [3]. The presence of macrophages is the main factor involved in emphysema because they release inflammatory mediators such as TNF-α, IL-6, and MMPs at the injury site. The persistence of macrophages at the injured area has been thought to be associated with the development of chronic inflammation and lung emphysema. We previously demonstrated that CSPGs were upregulated in a murine model of the lung with bleomycin-induced pulmonary fibrosis. Deposited CSPGs retained macrophages in fibrotic interstitium in a CD44-dependent manner. In addition, digestion of CSPGs by CSPG-digesting enzyme ChABC increased survival and reduced fibrosis by inhibiting persistent macrophage accumulation [4]. In this study, we demonstrated that reduced deposited C6SPG by CHST3 siRNA treatment impaired the accumulation of macrophages in BALF and in lung parenchyma (Figs. 5 and 6). Moreover this treatment decreased MLI and prevented an increase in lung volume and lung compliance (Figs. 1 and 2). Therefore, the present study indicates an important role of CSPGs for the development of emphysema induced by elastase injection.

CSPGs have been recognized as playing an important roles in cell adhesion, proliferation, tissue morphogenesis, neurite outgrowth, infections, and inflammation/leukocyte trafficking [26, 27]. Chondroitin sulfate (CS) chains are found as proteoglycan side chains in the extracellular matrices and at cell surface, and the main components of cartilage consisting of repeating disaccharide units sulfated either at the C-6 or the C-4 position of the GalNAc. Versican, which is large chondroitin sulfate proteoglycan, is a structural component of the ECM and a molecule that interacts with cells. In addition, Versican plays important roles in leukocyte adhesion and activation. In COPD, the modulation of versican influences elastic fiber deposition [28, 29]. CHST3, the gene encoding chondroitin 6-sulphotransferase-1, catalyzes modifying step of CS synthesis by transferring sulfate to the C-6 position of the GalNAc of chondroitin [30]. CHST3 is up-regulated in injured central nervous system (CNS) and inhibits axonal regeneration [31]. CHST3 is a key enzyme that mediates schwann cell migration during axonal growth [10]. In contrast, CSPGs in the inflammatory processes of various chronic inflammatory diseases in vivo have not been reported. To identify the in vivo role of CSPGs in a pulmonary emphysema model, we used the atelocollagen-mediated systemic delivery method for siRNA targeting CHST3. In the present study, we found upregulated expression of CHST3 mRNA after elastase challenge. Administration of CHST3 siRNA could reduce the CHST3 level in lung (Fig. 3). In addition, immunocytochemistry indicated deposited CSPGs, specially C6SPG were seen diffusely in the alveolar wall after elastase challenge. CHST3 siRNA successfully resulted in the limitation of CSPGs and C6SPG (Fig. 4).

During the early inflammatory phase (day 7), CHST3 siRNA reduced the number of F4/80^+^ macrophages in lung parenchyma and macrophages in BAL (Figs. 5 and 6). In addition, the expression of TNF-α and MMP-9 were suppressed significantly by CHST3 siRNA (Fig. 7). Based on previous reports, MMP-9 secretion is increased in alveolar macrophages from COPD patients compared to smokers without the disease [32]. MMP-9 transgenic mice developed significant air space enlargement associated with the loss of alveolar elastin [33]. Macrophages release a variety of inflammatory mediators including TNF-α and IL-1β as well as MMPs, including MMP-9 and MMP-12. MMP-9 production in human monocyte/macrophages is related to autocrine stimulation by TNF-α [34]. In addition, TNF-α can be released in vitro by the proteolytic action of MMPs, including MMP-9 [35]. MMP-9, produced by the activated macrophages, has the ability to cleave elastin with a majority of emphysema progressive. Elastin fragments, degradation of elastin by MMPs, promote the accumulation of monocytes from the circulation into lung tissue [36]. Thus, C6SPG directly regulates the accumulation of macrophages and the expression of TNF-α and MMP-9. In addition, Anti-inflammatory effect by C6SPG-synthesis block the feedback loops by elastin fragment.

During the late phase (Day 21), F4/80^+^ macrophages were still detected in the lung parenchyma, despite the decrease in mediators, TNF-α and MMP-9. This result suggests that the recruitment of macrophages in the late phase may be less dependent on elastin fragmation by MMP-9 than on other factors that may play a key role in the progression from inflammation to emphysema. We previously demonstrated that deposited CSPGs retained macrophages in fibrotic interstitium in a CD44-dependent manner in a bleomycin-induced pulmonary fibrosis model [4]. Thus, in this study, prolonged deposition of C6SPG might be responsible for retaining recruited macrophages within the interstitium. Blockage of C6SPG-synthesis prevents increased deposited C6SPG, which acted as a scaffold of macrophages, and diminishes accumulation of macrophages. Significant increase in MLI was already observed at day 7 and increased at day 21 in control mice. By contrast, CHST3 siRNA prevented the increase of MLI. Moreover, lung function by CHST3 siRNA was recovery to sham group.

CSPGs in the alveolar wall in COPD patients are negatively correlated to elastin and EBP [5]. CSPGs inhibit efficient repair by inhibiting re-synthesis of elastic fibers in alveoli of COPD patients. We reasoned that deposited CSPGs inhibit the elastin recovery from airway damage also in the elastase-induced pulmonary emphysema model [8, 9]. Elastic fiber in the alveolar wall of control siRNA reduced on day 7 and further on day 21. Although the reduction of elastic fiber in CHST3 siRNA was observed on day 7, CHST3 siRNA recovered the reduction of elastic fiber after day 21 (Fig. 8). Thus, CHST3 siRNA suppressed the progressive destruction of the elastin and rather, induced the lung recovery by promoting elastic repair.

**Fig. 9:**
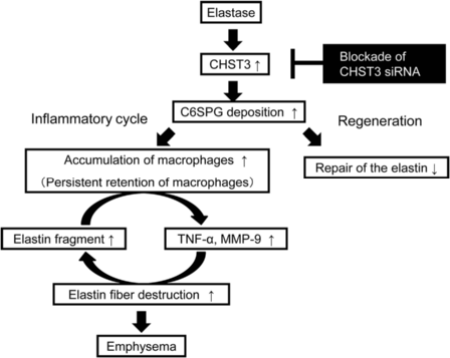
Schematic representation of the role of CHST3 in elastase-induced emphysema. In the inflammatory phase, CHST3-mediated C6SPG regulated accumulation of macrophages and the mediators, leading to elastic fiber destruction. In the regeneration phase, CHST3-mediated C6SPG inhibits repair of elastin. CHST3 siRNA diminishes accumulation of excessive macrophage and mediators and accelerates the elastin recovery

Based on these observations, we propose the role of CSPGs in elastase-induced pulmonary emphysema model (Fig. 9). In the inflammatory phase, CHST3-mediated C6SPG regulates macrophage recruitment and augments TNF-α and MMP-9 activities. These mediators lead to elastic fiber destruction with production of elastin fragment. Furthermore, these fragments in turn lead to the macrophage accumulation. CSPGs act as a scaffold of macrophages. The malignant cycle by elastin fragments contributes to emphysema progression. CHST3 siRNA breaks this cycle, resulting in amelioration from chronic inflammation to emphysema.

In the regeneration phase, CHST3-mediated C6SPG inhibits repair of elastin. CHST3 siRNA accelerates the elastin recovery.

One of the limitations of this study is that we did not evaluate the effect of cigarette smoke exposure. Cigarette smoke exposure models are more similar to human pulmonary emphysema and are scheduled in future.

COPD has been recognized as a systemic disease based on systemic inflammation [37]. In present study, we therefore used the systemic delivery method. It is known that a loss of CHST3 function results in human chondrodysplasia. Local administration of siRNA intratracheally may be more important than systemic administration.

## Conclusions

CHST3 siRNA treatment significantly reduced macrophage accumulation and the mediators. Furthermore, CHST3 siRNA accelerated the functional recovery from airway damage by repair of the elastin network. This finding that CHST3 siRNA prevents chronic lung inflammation and emphysema might be of use in the development of new therapeutic approaches for chronic inflammatory disease.

## Abbreviations

BALF: bronchoalveolar lavage fluid
C6SPG: chondroitin 6-sulfate proteoglycan
ChABC: chondroitinase ABC
CHST3: carbohydrate sulfotransferase 3
CNS: central nervous system
COPD: chronic obstructive pulmonary disease
CS: chondroitin sulfate
CSPG: chondroitin sulfate proteoglycan
Cst: static lung compliance
EBP: elastin-binding protein
GAG: glycosaminoglycan
GalNAc: N-acetylgalactosamine
IL-6: interleukin-6
MLI: mean linear intercept
MMP: matrix metalloproteinase
PPE: porcine pancreatic elastase
siRNA: small interfering RNA
TNF-α: tumor necrosis factor-α
